# What Drives Responses to Willingness-to-pay Questions? A Methodological Inquiry in the Context of Hypertension Self-management

**DOI:** 10.36469/9818

**Published:** 2016-10-27

**Authors:** Billingsley Kaambwa, Stirling Bryan, Emma Frew, Emma Bray, Sheila Greenfield, Richard J McManus

**Affiliations:** 1 Flinders Health Economics Group, School of Medicine Flinders University, Repatriation General Hospital, Daw Park, SA, Australia; 2 Centre for Clinical Epidemiology & Evaluation, Vancouver Coastal Health Research Institute and School of Population & Public Health University of British Columbia, Vancouver, BC, Canada; 3 Health Economics Unit, Institute of Applied Health Research University of Birmingham, Edgbaston, Birmingham, UK; 4 School of Psychology University of Central Lancashire, Preston, Lancashire, UK; 5 Primary Care Clinical Sciences, Institute of Applied Health Research University of Birmingham, Edgbaston, Birmingham, UK; 6 Primary Care Health Sciences, NIHR School for Primary Care Research University of Oxford, Oxford, UK

**Keywords:** methodological inquiry, self-management, hypertension, willingness to pay

## Abstract

**Background:** The use of economic evaluation to determine the cost-effectiveness of health interventions is recommended by decision-making bodies internationally. Understanding factors that explain variations in costs and benefits is important for policy makers.

**Objective:** This work aimed to test a priori hypotheses defining the relationship between benefits of using self-management equipment (measured using the willingness-to-pay (WTP) approach) and a number of demographic and other patient factors.

**Methods:** Data for this study were collected as part of the first major randomised controlled trial of self-monitoring combined with self-titration in hypertension (TASMINH2). A contingent valuation framework was used with patients asked to indicate how much they were willing to pay for equipment used for self-managing hypertension. Descriptive statistics, simple statistical tests of differences and multivariate regression were used to test six a priori hypotheses.

**Results:** 393 hypertensive patients (204 in the intervention and 189 in the control) were willing to pay for self-management equipment and 85% of these (335) provided positive WTP values. Three hypotheses were accepted: higher WTP values were associated with being male, higher household incomes and satisfaction with the equipment. Prior experiences of using this equipment, age and changes in blood pressure were not significantly related to WTP.

**Conclusion:** The majority of hypertensive patients who had taken part in a self-management study were prepared to purchase the self-monitoring equipment using their own funds, more so for men, those with higher incomes and those with greater satisfaction. Further research based on bigger and more diverse populations is recommended.

*JHEOR* 2016;4(2):158-71 | www.jheor.org | This is an Open Access article under the CC BY 4.0 license.

## BACKGROUND

Health systems worldwide are faced with the twin problems of expanding access to health care and controlling rapidly rising health care costs (global health spending is expected to rise from US$6.5 trillion in 2012 to US$9.3 trillion by 2018).[1,2] This therefore means that choices have to be made about how to distribute scarce health resources. Economic evaluation is an important technique to help decision-makers determine the relative value for money of service innovations in health care3 and is recommended for use by decision-making bodies internationally.[4] Understanding the value of new technologies and initiatives is a key component in economic evaluation.[3] One approach used for determining the monetary value of health benefits associated with such initiatives is willingness to pay (WTP) which is based on the contingent valuation methodology (CVM).[5-7] In the context of economic evaluation, heterogeneity in study populations may influence both costs and benefits[8] implying the possibility of WTP values varying across clinical populations. Decision makers internationally recommend incorporating subgroup analyses within economic evaluations.[9,10] Investigating characteristics that influence variations in WTP within economic evaluation would therefore help inform the comparisons of cost-effectiveness between subgroups especially when private cost perspectives are considered. In the literature, a number of characteristics have been identified to influence WTP for both health and non-health interventions.[3,11-28] This paper was designed to contribute to this methodological discussion using data collected from the first major randomised controlled trial of patient self-management in hypertension (TASMINH2).[29,30] We tested a priori hypotheses defining the relationship between WTP values for equipment used for self-management and a number of demographic and other patient factors. As far as we are aware, this is the first study to consider WTP for self-management in individuals with poorly controlled hypertension.

## METHODS

### The TASMINH2 Trial

The methodological details of the TASMINH2 trial have been reported elsewhere.[30] In brief, primary care physicians identified potential participants using electronic searches of clinical records from 24 general practices in the West Midlands, United Kingdom between March 2007 and May 2008.[31] Patients were eligible if they were aged 35-85, had a blood pressure at baseline of over 140/90 mmHg, were receiving treatment for hypertension with two or fewer antihypertensive drugs and were also willing to self-monitor and self-titrate medication. Patients randomised to the self-management arm were trained in the use of an automated sphygmomanometer (Omron 705IT, Omron Healthcare Europe, Hoofddorp, Netherlands) and related equipment to take and transmit blood pressure readings.[29] A colour traffic light system was used by these patients to code these readings: green (within target range), amber (above target but below safety limits) and red (very high or very low). Following an initial consultation with their primary care physicians at which they were given instructions on potential antihypertensive medications changes, patients could make such changes on the basis of their readings without needing to re-consult.[29] Participating primary care physicians were free to use any antihypertensive drug. Patients randomised to the usual hypertension care arm received an annual hypertension review as per UK national guidelines.[32,33]

### Willingness to Pay for Self-management Equipment (The Contingent Valuation Method)

WTP data were collected through self-completed questionnaires, with questions phrased within the framework of the CVM, a survey-based approach for eliciting individuals' monetary valuations for use in cost-benefit analysis.[34] Within this methodology, individuals are asked to consider a hypothetical scenario depicting the existence of an imaginary market for the benefits or losses of a health care programme or 'good'.[5] Using different design instruments, individuals are asked to state their WTP to reflect a welfare gain or willingness to accept (WTA) in compensation for a welfare loss.[5,35] The amount an individual is WTP or WTA is assumed to be an estimate of the perceived value the individual places on both the health and non-health consequences of the programme or good.[36]

## Outline and Administration of the WTP Survey Questionnaire

In this study, WTP values were elicited using open-ended questions while other information (employment status, household income level and which attributes of blood pressure machines were considered important) was collected using closed questions (Appendix). The open-ended question format was used because it does not introduce range or starting-point biases and it can also be highly statistically efficient compared to discrete choice formats.[37] The questionnaire was made up of a number of parts: the first described the use of the self-management within the TASMINH2 trial and presented the purpose of the questionnaire as wanting to establish the value participants placed on self-management equipment. Next, the questionnaire asked whether or not respondents owned a blood pressure machine at the time of the study, and if they did, where, when, and why they had bought the machine. In the subsequent section, information on the costs of blood pressure equipment in high street shops and pharmacies was presented and 'basic' equipment that allowed only the measuring of blood pressure readings, but not the transmission of these readings to physicians, was distinguished from 'advanced' equipment that enabled patients to take and transmit readings. This information was presented to all trial participants (regardless of whether they had undertaken self-management in the trial) to ensure that everyone had the same 'reference value' for the equipment prior to providing WTP values. Retail prices in 2008 (adjusted to 2015 prices) of between £12 and £93 were presented for basic equipment while the range for advanced equipment was £93 to £185. Open-ended questions on the maximum amount of money respondents would be WTP to buy blood pressure equipment (separate responses elicited for basic and for advanced equipment) and reasons for these WTP valuations followed. The last part of the questionnaire solicited information on employment status and household income levels. The questionnaire was self-completed by trial participants in both the intervention and usual care arms during the final follow-up session at 12 months.

## A priori Hypotheses

This section considers potentially relevant factors for the WTP of self-management of hypertension before summarising the a priori hypotheses tested.

Familiarity or prior direct experience with a product being valued has been shown to positively influence WTP for that product.[11-14] However, Bergmo and Wangberg[38] found that patients with a history of communicating electronically with their general practitioner valued this communication lower than those that did not have such a history. Similarly, Callan and O'Shea showed a negative relationship between WTP amounts for telecare programmes for informal care provision and high levels of technical proficiency gained from usage of such programmes.[15] Another study found that experience of using short messaging service (SMS) did not have any impact on the WTP for SMS health reminders.[16]

Age has also shown to have a negative effect on WTP for access to telemedicine in one study[17] but a positive one on WTP for SMS health reminders.[16] Other studies have however shown that age[18] and gender[16,19] do not significantly predict WTP for telehealth communication.

Generally in economics, a positive correlation between income and WTP has been postulated.[3,20,21] This relationship has also been seen when assessing the WTP of a health consultation service involving the use of a sphygmomanometer within a tele-conference system[22], for telecare programmes designed to support independent living[15] and for computerisation of clinical services.[23] Gender also appears to influence individuals' preferences for telecare programmes with recent findings indicating that men are WTP more than women[15] but this may be a result of an income effect with men generally associated with higher income.

A positive (negative) correlation between disease severity (good health status) and WTP for a service or therapy that offers health improvement in that disease has been shown in the literature.[21,24,25] Stephen et al[39] depicted a positive relationship between moderate dementia severity and WTP for telecare among carers but Viers et al[26] found positive correlation between current good health status and WTA videoconferencing amongst urological patients.

Cross et al[24] showed that patient satisfaction has a positive impact on the WTP for hip and knee joint replacement surgery for osteoarthritis. In some instances, patient satisfaction has actually been equated with WTP.[24,40] Perceived usefulness was also revealed as one of the predictors that plays an important role in perceptions of home telemedicine services among older adults.[28] Ogasawara and Abe also found that individuals that had a 'willingness to use tele-health consultation service' provided higher WTP values for it.[22]

Based on the relationships revealed in the studies above, and while controlling for a number of other patient characteristics, we sought to test the hypotheses that higher WTP values for self-management equipment were associated with: (i) familiarity with, or prior experience of using, the equipment (ii) males (iii) younger patients (iv) higher household incomes (v) deterioration (elevation) in blood pressure health outcomes/severity of hypertension as measured by the change in blood pressure over 12 months and (vi) higher satisfaction with self-monitoring (revealed through content analysis5 of the reasons given for the WTP valuations).

### Data Analysis

Data collected through WTP questionnaires were linked to data on other patient characteristics gathered as part of the main TASMINH2 trial including age, gender, quality of life status, blood pressure readings, trial arm, ethnicity, occupation and past medical history. Generation of descriptive statistics (medians, ranges, means and standard errors) and simple statistical tests of differences between groups of interest that took into account the distributional nature of the variables in the dataset (Kruskal Wallis and Pearson chi-square tests) were carried out.[41] Using these tests, we determined whether there were any differences between responders and non-responders to the WTP questionnaire, between those that provided zero WTP values and those that gave positive WTP valuations. A generalised linear regression modelling (GLM) approach[42] was used to test the six a priori hypotheses where WTP continuous values were the dependent variables. The models controlled for a number of factors including past medical history of Coronary Heart Disease (CHD) and Diabetes[29]; Body Mass Index (BMI)[43], Index of Multiple Deprivation (IMD)[44], baseline EuroQol EQ-5D 3 level (EQ-5D-3L) score[45] and reasons for WTP valuations. The modified park test suggested by Manning[42] was used to guide the choice of the GLM distribution and link to use in these regression analyses. GLM was chosen so as to deal with the twin problems of heteroscedasticity and non-normality.[42] Multiple imputation[46], based on an iterative Markov chain Monte Carlo method premised on a multivariate normal regression[47], was used to account for missing values and the regressions were therefore run on both complete case and multiply imputed datasets and the results compared. All costs were inflated to 2015 UK£ figures, and Stata version 14.0 software was used.[47]

## RESULTS

### Sample Characteristics

The final complete case sample in TASMINH2 trial (Table 1) was made up of 480 patients with a mean age of 69 years mean, baseline systolic blood pressure of 152 mmHg, and mean baseline EQ-5D-3L score of 0.81. On average over a 12-month period, the total sample registered QALY gains of 0.01 and reductions in blood pressure of 14mmHg while their mean baseline IMD and BMI scores were 17 and 30kg/m2, respectively. The majority were white (96%), married (75%), were or had been employed in a professional or skilled job (80%), had household incomes of £42,978 or less per annum (76%), non-smokers (93%), and without a medical history of diabetes (93%), CHD (90%), or CVD (96%).

As also shown in Table 1, a total of 393 patients answered the WTP questionnaire representing a response rate of 75% of individuals initially recruited to the trial (n=527) and 82% of those that attended final follow-up (n=480). The results show that there were no statistically significant differences between individuals that responded to the WTP questionnaire and those that did not answer the questionnaire (non-responders) except in terms of the IMD (higher for non-responders) and the trial arm a patient was in (ie: a higher proportion of individuals in the self-management arm completed the WTP questionnaire). Of the 393 that answered the WTP questionnaire, 58 (15%) were 'zero responders' ie they provided zero WTP values for self-management equipment. Table 1 shows that the only statistically significant differences in patient characteristics between these zero responders and patients who provided positive WTP values were in terms of age (with the former being older, ie: mean age of 71 vs 68 years) and mean QALYs gained after 12 months (higher in patients with positive WTP values ie 0.02 vs -0.05). Just over one-quarter (105) reported having had someone recommend that they purchase a blood pressure machine, with family or friends and GPs being the top recommenders (in 41 and 27% of all cases, respectively).

### WTP Amounts

As expected, and in accordance with economic theory, WTP values for both types of equipment were right skewed (Figure 1). Table 2 presents the maximum amounts respondents were WTP for self-management equipment compared against: (i) average retail prices of similar machines that were obtaining in high street shops and pharmacies at the time of the study and (ii) purchase prices of blood pressure machines as reported by those who owned similar machines at the time of the study. Of the 216 (55%) of respondents who reported owning a similar blood pressure machine at the time of the study, 212 provided valid purchase prices (mean price of £43 per machine). The WTP values for the newer advanced equipment were higher than those for basic equipment that participants had prior experience of using (£85 vs £58). Compared to the purchase prices, the WTP mean figures for basic equipment were on average about 1.4 times higher while those for advanced equipment were about twice as high. As also shown in Table 2, purchase prices positively influenced WTP values with a statistically significant positive correlation ($p < 0.05$) between WTP amounts and purchase prices of similar blood pressure machines owned by patients with correlation coefficients ranging from 0.52 to 0.64 (basic equipment) and from 0.27 to 0.37 (advanced equipment).

**Table 1.** Baseline Patient Characteristics for Total Sample, WTP Questionnaire Responders and WTP Questionnaire Non-responders

| Patient Characteristics | Total sample[a] (n = 480) | Responders[a] (n = 393) | Non-responders[a] (n = 87) | p-value[b] | Zero-responders[a] (n = 58) | +ve WTP responders[a] (n = 335) | p-value[b] |
|---|---|---|---|---|---|---|---|
| Mean Age | 68.46 [8.75] | 68.72 [8.61] | 67.31 [9.29] | 0.20 | 71.20 [9.67] | 68.29 [8.36] | 0.02 |
| Mean baseline EQ-5D-3L[c] score | 0.82 [0.23] | 0.81 [0.24] | 0.85 [0.22] | 0.09 | 0.83 [0.17] | 0.81 [0.25] | 0.93 |
| Mean 12-month QALY[d] gain | 0.01 [0.24] | 0.01 [0.25] | -0.03 [0.23] | 0.06 | -0.05 [0.22] | 0.02 [0.25] | 0.04 |
| Mean systolic blood pressure reading | 151.98 [11.77] | 151.79 [11.60] | 152.84 [12.53] | 0.64 | 153.56 [13.48] | 151.48 [11.24] | 0.49 |
| Mean 12-month blood pressure elevation | -14.41 [18.36] | -14.33 [18.18] | -14.78 [19.24] | 0.67 | -13.28 [20.84] | -14.52 [17.71] | 0.67 |
| Mean index of multiple deprivation[e] | 17.05 [13.62] | 15.83 [12.44] | 22.56 [17.07] | <0.01 | 17.32 [12.48] | 15.57 [12.43] | 0.14 |
| Mean body mass index (kg/m2)[e] | 29.77 [5.61] | 29.73 [5.77] | 29.95 [4.85] | 0.46 | 26.69 [5.82] | 29.73 [5.77] | 0.92 |
| Female gender n (%) | 255 (53) | 210 (53) | 45 (52) | 0.08[f] | 34 (57) | 176 (53) | 0.39[f] |
| Intervention trial arm n (%) | 234 (49) | 204 (52) | 30 (34) | <0.01[f] | 26 (45) | 178 (53) | 0.24[f] |
| Ethnicity n (%) | | | | | | | |
| White | 461 (96) | 378 (96) | 83 (95) | | 57 (98) | 321 (96) | |
| Black | 7 (1) | 5 (1) | 2 (2) | | 0 | 5 (1) | |
| Asian | 10 (2) | 8 (2) | 2 (2) | | 1 (2) | 7 (2) | |
| Other | 2 (1) | 2 (0.5) | 0 | 0.80[f] | 0 | 2 (1) | 0.74[f] |
| Marital status=married, n (%) | 362 (75) | 299 (76) | 63 (72) | 0.47[f] | 44 (76) | 255 (76) | 0.97[f] |
| Occupation | | | | | | | |
| Professional/managerial/ technical | 219 (46) | 180 (46) | 39 (45) | | 19 (33) | 161 (48) | |
| Skilled manual and non-manual | 163 (34) | 132 (34) | 31 (36) | | 24 (41) | 108 (32) | |
| Partly skilled and unskilled | 30 (6) | 23 (6) | 7 (8) | | 5 (9) | 18 (5) | |
| Unemployed/ unwaged | 68 (14) | 58 (15) | 10 (11) | 0.76[f] | 10 (17) | 48 (14) | 0.18[f] |
| Household Income[g] | | | | | | | |
| Less than £28 405 | 169 (51) | 166 (951) | 3 (75) | | 15 (60) | 151 (50) | |
| £28 405 -£42 978 | 83 (25) | 82 (25) | 1 (925) | | 5 (20) | 77 (26) | |
| £42 979 - £53 305 | 39 (912) | 39 (12) | 0 | | 4 (16) | 35 (12) | |
| £57 306 - £71 631 | 18 (5) | 18 (6) | 0 | | 0 | 18 (6) | |
| More than £71 631 | 21 (6) | 21 (6) | 0 | 0.84[f] | 1 (4) | 20 (7) | 0.58[f] |
| Current smoker n (%) | 33 (7) | 26 (7) | 7 (8) | 0.63[f] | 3 (5) | 23 (7) | 0.63[f] |
| Past medical history of diabetes n (%) | 35 (7) | 27 (70) | 8 (9) | 0.45[f] | 2 (3) | 25 (7) | 0.26[f] |
| Past medical history of CHD[h] n (%) | 46 (10) | 35 (9) | 11 (13) | 0.28[f] | 6 (10) | 29 (9) | 0.68[f] |
| Past medical history of CVD[i] n (%) | 21 (4) | 19 (5) | 2 (2) | 0.30[f] | 4 (7) | 15 (4) | 0.43[f] |

[a]Figures in [ ] are standard deviations; [b]p-value from Kruskal Wallis test for differences between responders & non-responders, unless otherwise stated; [c]EQ-5D-3L: EuroQoL 5 Dimensions 3 Level instrument; [d]QALY: Quality-adjusted life years; higher QALY scores imply lower disease burden; [e]Higher index of multiple depression score implies more deprivation; higher body mass index score = higher amount of tissue mass; [f]P-value from Chi-Square test; [g]Missing data for household income, n=326; (income ranges inflated from 2008-2015 UK£ prices); [h]CHD:coronary heart disease; [i]CVD: cerebrovascular disease

**Figure 1.** Distribution of Willingness to Pay (WTP) Values for Basic and Advanced Equipment

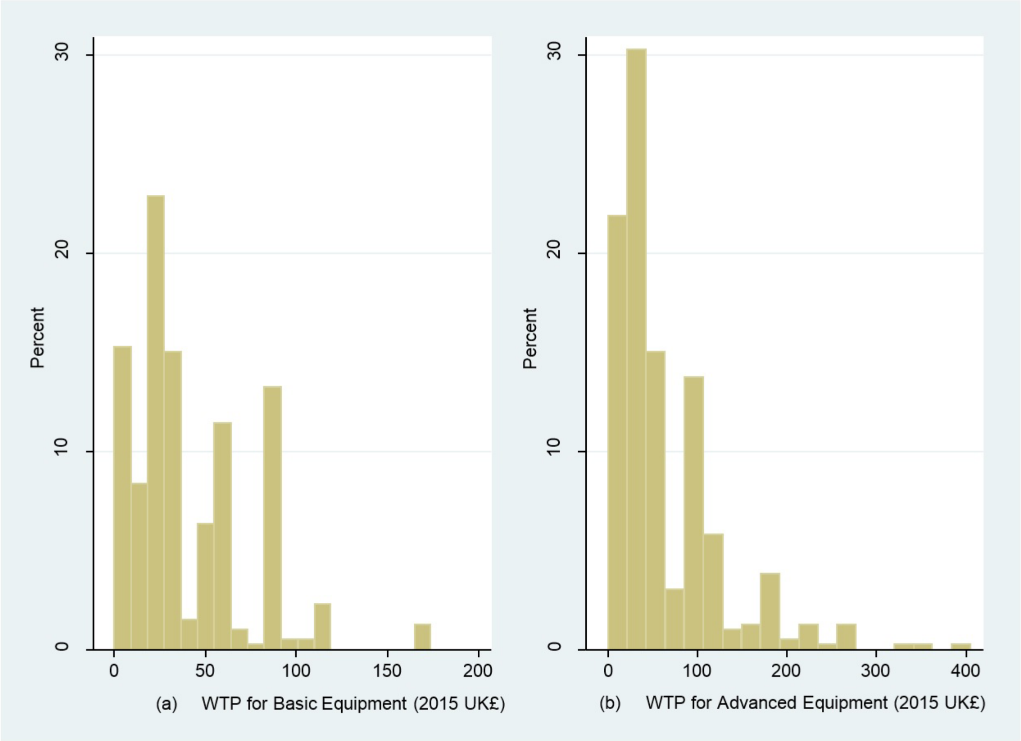

Distribution of willingness to play values, measured in UK pounds 2015 prices, for basic (a) and advanced (b) equipment. They show that willingness to pay values for both types of equipment were right skewed.

**Table 2.** WTP Amounts, Blood Pressure Machine Purchase and Retail Prices (2015 UK£)[a]

| | Trial Arm | Basic Equipment | Advanced Equipment |
|---|---|---|---|
| WTP amounts (UK£) | Intervention arm[b] | 57 (40); 43[28, 72] | 83 (72); 61[28, 107] |
| | Usual care arm[b] | 59 (38); 43[28, 72] | 89 (82); 64[28, 107] |
| | Entire sample[b] | 58 (38); 43[28, 72] | 85 (75); 64[28, 107] |
| Median (range) retail prices (UK£) | Entire sample | 61 (14, 107) | 322 (107, 215) |
| Blood pressure machine purchase prices[c] (UK£) | Intervention arm | 42 (43); 25[15, 56] | |
| | Usual care arm | 43 (44); 23[15, 62] | |
| | Entire sample | 43 (43); 23[15, 58] | |
| **Correlationd between positive WTP value amounts and blood pressure purchase prices[e]** | | | |
| Correlation coefficients (p value) | Intervention arm[b] | 0.52 ($p < 0.01$) | 0.27 ($p = 0.01$) |
| | Usual care arm[b] | 0.64 ($p < 0.01$) | 0.37 ($p < 0.01$) |
| | Entire sample[b] | 0.58 ($p < 0.01$) | 0.32 ($p < 0.01$) |

[a]Figures are mean (standard deviation); median [quartile1, quartile 3] ,unless otherwise stated
[b]n = 178 for intervention arm, 157 for usual care arm and 335 for entire sample
[c]n = 104 for intervention arm, 108 for usual care arm and 212 for entire sample
[d]Spearman correlation
[e]These are purchase prices for blood pressure machines owned by patients at the time of the study

**Table 3.** GLM Regression Results (Predictors of Willingness-to-pay amounts)

| | Basic Equipment | | | Advanced Equipment | | |
|---|---|---|---|---|---|---|
| **Patient Characteristics** | **Coef.** | **Std. Err.** | **P-value** | **Coef.** | **Std. Err.** | **P-value** |
| Main Effects | | | | | | |
| Gender (1=Female, 0=Male) | -0.27 | 0.11 | 0.01 | -0.29 | 0.13 | 0.03 |
| Age (continuous variable) | 0.01 | 0.00 | 0.29 | -0.01 | 0.01 | 0.12 |
| Past medical history of Coronary Heart Disease (1=Yes, 0=No) | -0.24 | 0.13 | 0.06 | -0.18 | 0.16 | 0.28 |
| Past medical history of diabetes? (1=Yes, 0=No) | -0.26 | 0.15 | 0.09 | -0.17 | 0.19 | 0.36 |
| Body Mass Index in $kgm^2$ (continuous variable) | 0.01 | 0.01 | 0.37 | 0.01 | 0.01 | 0.35 |
| Mean Index of Multiple Deprivation (continuous variable) | 0.01 | 0.00 | 0.08 | 0.01 | 0.00 | 0.05 |
| Mean 12-month blood pressure elevation (continuous variable) | 0.00 | 0.00 | 0.17 | 0.00 | 0.00 | 0.11 |
| Baseline EQ-5D-3L[a] score (continuous variable) | -0.19 | 0.16 | 0.25 | 0.00 | 0.20 | 0.98 |
| Trial Group (1 = intervention, 0 = control) | 0.02 | 0.04 | 0.68 | -0.01 | 0.05 | 0.78 |
| Household income/annum (categorical variable)[b] | | | | | | |
| < £23,700 (Ref.) | | | | | | |
| £23,701 - £35,800 | -0.08 | 0.13 | 0.57 | 0.14 | 0.17 | 0.41 |
| £35,800 - £47,800 | 0.40 | 0.17 | 0.02 | 0.51 | 0.21 | 0.02 |
| £47,801 - £59,800 | 0.14 | 0.21 | 0.51 | 0.33 | 0.26 | 0.20 |
| > £59,800 | -0.01 | 0.19 | 0.95 | 0.11 | 0.24 | 0.64 |
| Reason for WTP[c] valuation (1 = Amount reflects ability to pay, 0 = Otherwise) | 0.17 | 0.10 | 0.10 | 0.05 | 0.12 | 0.66 |
| Reason for WTP[c] valuation (1= Amount is a reasonable value, 0 = Otherwise) | 0.14 | 0.10 | 0.17 | 0.22 | 0.12 | 0.07 |
| Reason for WTP[c] valuation (1= Amount reflects satisfaction with equipment, 0 = Otherwise) | 0.41 | 0.13 | 0.00 | 0.52 | 0.16 0 | .00 |
| | | | | | | |
| **Interactions** | | | | | | |
| Gender (Female) x Household income ('<£23,700') (Ref) | | | | | | |
| Gender (Female) x Household income ('£23,701 - £35,800') | 0.34 | 0.19 | 0.08 | 0.28 | 0.24 | 0.25 |
| Gender (Female) x Household income ('£35,800 - £47,800') | -0.25 | 0.24 | 0.30 | -0.06 | 0.31 | 0.83 |
| Gender (Female) x Household income ('£47,801 - £59,800') | -0.34 | 0.34 | 0.31 | -0.12 | 0.42 | 0.78 |
| Gender (Female) x Household income ('> £59,800') | 0.65 | 0.33 | 0.05 | 0.20 | 0.41 | 0.63 |
| | | | | | | |
| Constant | 3.35 | 0.48 | 0.00 | 4.49 | 0.58 | 0.00 |
| | N = 422; R-squared = 0.12 | | | N = 422; R-squared = 0.14 | | |

[a] EQ-5D-3L = EuroQoL 5 Dimensions 3 Level instrument
[b] Household income categories inflated to UK £ 2014 prices
[c] WTP = willingness to pay

### Factors Affecting WTP Valuations

The results of the GLM regression model are shown in Table 3. The coefficients for the natural log of predicted WTP valuations from the modified park test for the basic and advanced equipment GLM models were 1.52 and 1.01, respectively, suggesting that a Poisson distribution (and log link function) was best suited for modelling all the valuations. As the GLM results based on multiple imputed data and those based on complete cases were not significantly different, only results based on the former are presented. Higher WTP amounts for basic equipment were associated with being male, a higher household income and satisfaction with the basic equipment (i.e. the perception that the use of the equipment would lead to immediate or future clinical and economic benefits). The effect that gender had on WTP did not differ significantly according to household income. There was also a trend for higher WTP values amongst individuals who did not have a past medical history of CHD ($p = 0.06$). Similar to WTP for basic equipment, higher WTP values for advanced equipment were associated with being male (relationship did differ according to household income), a higher household income and satisfaction with advanced equipment while a positive trend was seen between these values and IMD ($p = 0.054$). Unlike WTP for basic equipment, there was a trend for higher WTP values for advanced equipment to be associated with the perception that WTP valuations were fair, acceptable or reasonable ($p = 0.07$). In both models, WTP did not differ according to trial group (previous versus no experience of undertaking self-management), age or changes in blood pressure.

## DISCUSSION

### Statement of Principal Findings

On the basis of the number of individuals who provided positive WTP values, this study shows that the majority of hypertensive patients were prepared to purchase the self-management equipment using their own funds. Patients were willing to pay nearly 50% more for advanced equipment than they were for basic equipment and this may be a reflection of the fact that the former includes telemonitoring which allows for better communication with their doctor. The positive relationship between mean QALYs gained after 12 months and WTP values gives further support to the validity of using WTP to value benefits of using this equipment. This relationship has been seen elsewhere.[48-51] Of the 6 hypotheses tested, 3 were accepted regardless of the equipment valued: higher WTP values were associated with being male, higher household incomes and satisfaction with the equipment. These results correspond with previous research in this area.[3,15,20-23,27,28] Prior experience of undertaking self-management within the trial, age and deterioration in blood pressure outcomes over the trial period did not have an effect on WTP. Though these relationships were not as hypothesised, other research has shown similar results[16,18,52] and a number of factors may explain these findings.

First, while prior experience and familiarity with technology may lead to higher WTP values for that particular technology, research also shows that this experience can be countered by perceptions of potential benefits, such as improved functioning and efficiency, from alternative technology.[14] In addition, more than half of the patients in both TASMINH2 trial arms reported having used blood pressure machines prior to taking part in the trial. These patients may, therefore, have already formed perceptions about the equipment that were not altered by their use of it in the trial. Exposure to the equipment during the course of the trial did not therefore lead to revised valuations though the trend towards a higher WTP for advanced equipment suggests that this type of equipment may have been valued slightly but not statistically higher. Although some research has shown that younger respondents tend to have a more positive attitude towards, and are therefore WTP more for, new technology[14], age was not revealed to be a significant predictor of WTP in this study. This may be because of a lack of significant age differentiation in our sample of predominantly older people (i.e. over 90% were aged

55 years or older). Lastly, a change in blood pressure was not a significant predictor of higher WTP. On average, all individuals in the TASMIH2 trial experienced significant blood pressure reductions (mean values of 17.6 and 12.2mmHg in the intervention and control groups, respectively).[29] It is therefore possible that while individuals who experienced this reduction may have been WTP for self-management equipment, the differences in reduction between groups was not significant enough in itself to lead to statistically higher WTP values. Furthermore, as blood pressure is largely asymptomatic, individuals may not have distinguished between differences in blood pressure. Other research has shown that controlling for other factors such duration of illness may help explain such relationships better.[53]

Our finding of a positive relationship between purchase prices and WTP supports the hypothesis that previous and present prices positively affect internal reference prices, or a respondent's expectation of a reasonable price level.[54] Similar results have been seen in other research within the healthcare sector.[55] Overall, hypertension patients that responded to the WTP questionnaire were no different in terms of patient characteristics to those who did not respond to the questionnaire except with regards to the IMD and the trial arm they were in. Given the positive link shown in the literature between household income and WTP[3], it is surprising that deprived individuals were more willing to respond and give a valuation of self-management equipment. Most deprived individuals in our sample, however, were older and therefore at a greater risk of adverse health events and may thus have been more willing to participate in the WTP study compared with younger respondents. It is also possible that those in the intervention arm were more motivated than their counterparts in the usual care arm to participate in this sub study as they were more likely to have already experienced some benefits from self-management.

### Strengths and Limitations

This study used data from the first major randomised controlled trial of self-management which had high levels of follow up and data capture.[29] The study is also, as far as we are aware, the first to consider WTP for self-management in individuals with poorly controlled hypertension. It was performed on a population that included individuals who had used the self-management equipment and those that had not, all drawn from primary care, and was therefore representative of the general hypertensive population. In our study, we presented retail price ranges for the self-management equipment and this information may have influenced patients' WTP valuations. In addition, about half of the sample were familiar with the equipment and had previously spent their own money on self-monitoring equipment. Some, however, argue that including information which makes the contingent market more realistic leading to valuations that bear some relation to actual values.38 We nevertheless included open-ended format questions which respondents used to provide actual WTP valuations which may have negated the bias resulting from having the retail prices' information with the survey in the first place.

### Comparisons with Other Studies

This is, as far as we know, the first WTP study assessing what value hypertensive patients placed on self-management equipment. Other related studies based on WTP have been reported in the literature: a Japanese study that analysed factors affecting WTP for cardiovascular disease-related medical services found WTP for hypertension was significantly higher in married males and the group with symptoms but was not associated with income.[56] Bergmo and Wangberg assessed Norwegian patients' WTP for electronic communication with their general practitioners and found that more than half of their study population were WTP for such communication with older patients associated with higher WTP values.[38] In a study that examined WTP for antihypertensive care based on a population of hypertensive patients in a large, staff-model, managed care organisation, Ramsey et al[57] found that WTP values were significantly associated with higher income levels and the perception that the antihypertensive therapy was beneficial while current perceived status, age, gender and education were not significant.

## CONCLUSIONS

This study adds to the growing literature on WTP for self-management for people with hypertension and particularly formally tests hypothesised relationships between WTP values in hypertension self-management and a number of patient characteristics. Gender (male), higher household incomes and satisfaction with equipment were shown to be the common predictors of higher WTP values regardless of the equipment evaluated. As there was a lack of significant variation within variables related to age, changes in blood pressure and previous experience of using self-management equipment, we recommend that future research be applied to bigger and more diverse study populations.

## Figures and Tables

**Figure:** Supplementary Content

